# Objective Measurements of Nasal Obstruction and Eustachian Tube Function in Pregnant Women

**DOI:** 10.3390/jcm13092671

**Published:** 2024-05-02

**Authors:** Alicja Grajczyk, Krystyna Sobczyk, Justyna Zarzecka, Ewa Barcz, Karolina Dżaman

**Affiliations:** 1 Department of Otolaryngology, Collegium Medicum, Faculty of Medicine, Cardinal Stefan Wyszyński University, Międzylesie Specialist Hospital, 01-938 Warsaw, Poland; alicja.grajczyk@gmail.com; 2 Department of Otolaryngology, Centre of Postgraduate Medical Education, Marymoncka 99/103, 01-813 Warsaw, Poland; krsobczyk@gmail.com; 3 Department of Gynecology and Obsetrics, Collegium Medicum, Faculty of Medicine, Cardinal Stefan Wyszyński University, Międzylesie Specialist Hospital, 01-938 Warsaw, Poland; jmgilewska@gmail.com (J.Z.); e.barcz@uksw.edu.pl (E.B.)

**Keywords:** rhinitis, Eustachian tube edema, pregnancy

## Abstract

**Background**: Hormonal changes during pregnancy have a substantial effect on the swelling of the mucous membrane in the nasal cavity, resulting in rhinitis and Eustachian tube dysfunction. The aim of the study was to assess subjective and objective changes in nasal cavity and Eustachian tube patency in the third trimester of pregnancy. **Materials and Methods**: The study group included fifty 18–41 year-old women in the third trimester of pregnancy. The control group consisted of 25 females aged 25 to 31 who were not pregnant. The Eustachian tube and nasal cavity patency examination was carried out using a rhinomanometer, a tympanometer and a SNOT-22 Questionnaire. **Results**: The SNOT-22 showed that subjective nasal obstruction was assessed as strong or worse by 42% of the study group, which was significantly higher than in the control group and confirmed with rhinomanometry. A total of 68% of the study group reported a subjective ear fullness which correlated with the week of pregnancy. The tympanometry showed that all pregnant women exhibited a type A tympanogram, but tympanometry values decreased at higher weeks of pregnancy, and statistical analysis confirmed a significant difference between the study group and the control group in tympanometry for both ears. **Conclusions**: The results highlight the substantial impact on both subjective and objective measurements of nasal and Eustachian tube patency. Almost every woman in the third trimester of pregnancy experiences nasal obstruction, and nearly 70% struggle with ear fullness. Recognizing and addressing these challenges are pivotal for ensuring the well-being of pregnant individuals and enhancing the quality of antenatal care.

## 1. Introduction

The distinctive nature of female sex hormones lies in their cyclic variations observed during pregnancy, menopause, and the menstrual period. These variations in estrogen and progesterone levels lead to physiological changes in the female body [1]. Pregnancy, marked by hormonal shifts, affects various organs, including the nasal cavity and the otological system. Additionally, sex hormones exert influence on the central nervous system of pregnant women [2].

**Figure 1:**
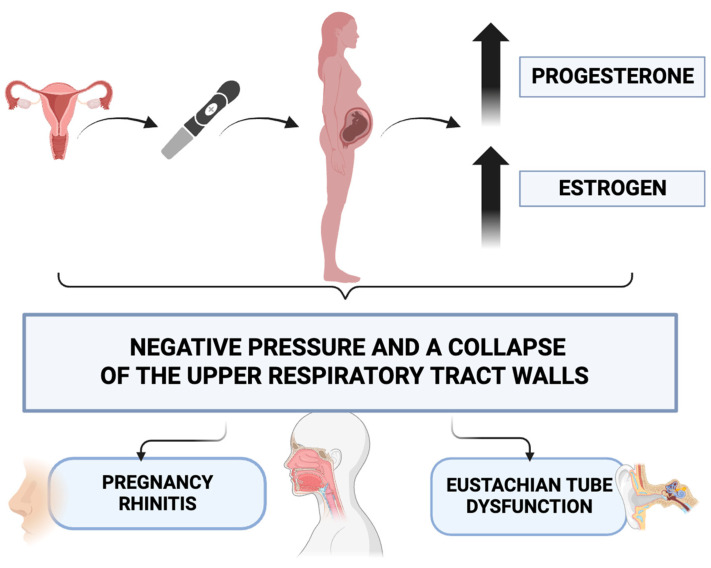
Physiological changes occurring during pregnancy, affecting the upper respiratory tract.

Pregnancy entails significant transformations in a woman’s body, with the majority of these changes posing no harm to the mother or fetus. However, some alterations can give rise to pathological manifestations. Levels of estrogen and progesterone undergo a continuous rise throughout pregnancy, reaching their highest point in the final trimester, and subsequently decline shortly after childbirth [3]. Estrogen and progesterone are responsible for the increased permeability of vessel walls and the relaxation of smooth muscles in vessels, causing an increase in negative pressure and a collapse of the upper respiratory tract walls, and certain clinical manifestations are observed in the head and neck region [4]. These clinical symptoms encompass issues such as epistaxis, rhinitis, otological disorders, and alterations in voice [5,6,7]. The aforementioned physiological changes occurring during pregnancy, affecting the upper respiratory tract, contribute to a deterioration in the quality of life for pregnant women (Figure 1). Nasal obstruction and torus tubarius swelling result in pregnancy rhinitis and secondary Eustachian tube dysfunction, leading to hearing loss and discomfort related to ear congestion caused by negative pressure in the middle ear.

Nasal obstruction or rhinitis during pregnancy has been acknowledged as a distinct condition for an extended period of time. It occurs in approximately 5–32% of pregnant women, typically emerging towards the end of the first trimester, and may persist until delivery or a few weeks thereafter. This condition is characterized by clear rhinorrhea, and clinical examination reveals edematous nasal mucosa [8]. The root cause lies in the heightened interstitial fluid volume observed during pregnancy, exacerbated by the direct impact of estrogen and progesterone on nasal mucosa, leading to increased vascularity and mucosal edema. The overactivity of the parasympathetic system in pregnant women may contribute as an additional factor [9].

Dysfunction of the Eustachian tube is estimated to impact around 5–30% of pregnant women, exhibiting variable symptomatology that may involve either tubal obstruction or patulous Eustachian tubes [10]. The onset of signs and symptoms typically occurs after the first trimester, and the nature of the dysfunction determines the specific manifestations. Women experiencing tubal obstruction may report sensations of ear blockage or popping, along with muffled sounds. Severe cases may lead to the development of serous otitis media (SOM). Conversely, those with patulous tubes often display intermittent symptoms such as autophony and a roaring sensation in the ears synchronized with respiration. These symptoms are more pronounced in the upright position or during exercise. The underlying pathophysiology is linked to mucosal edema [11,12].

It has to be mentioned that the majority of otorhinolaryngological disturbances observed in pregnant women are benign and typically resolve on their own. That is why they are often downplayed by doctors, even though they significantly reduce the quality of life for pregnant women. Therefore it remains crucial for otorhinolaryngologists to assess and manage these complications, providing reassurance to those patients. Moreover, medical practitioners and gynecologists should be prepared to anticipate and address these manifestations to offer reassurance and ensure the safe management of such symptoms [1,3,13].

A comprehensive understanding of both the physiological and pathological aspects of pregnancy allows for the safe and effective management of otorhinolaryngological manifestations in pregnant women. Therefore, the aim of the study was to assess subjective and objective changes in nasal cavity and Eustachian tube patency in the third trimester of pregnancy.

## 2. Materials and Methods

**Figure 2:**
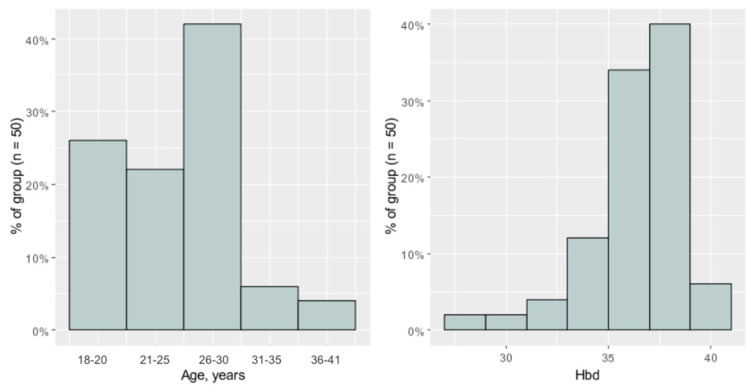
Histograms of age and Hbd (week of pregnancy) in the study group.

The study group included fifty 18–41 year-old females (av. 27.04 ± 5.17 years) in the third trimester of pregnancy (28th–41st week of pregnancy, av. 37 weeks) examined at the Department of Otolaryngology in Międzylesie Specialist Hospital in Warsaw, Poland. (Figure 2).

The control group consisted of 25 healthy non-pregnant women and medical students aged 24 to 32 (mean ± SD: 26.00 ± 2.58, the median is 25).

Patients with severe pathologies that could influence nasal patency, e.g., deformations of the nasal septum and external nose, or inflammatory and cancerous lesions revealed in the ENT examination, were excluded from the study.

Each patient participating in the research project underwent ENT examination with anterior and posterior rhinoscopy, upper respiratory tract fibroscopy and otoscopy.

To assess changes in nasal cavity and Eustachian tube patency, rhinomanometry and tympanometry were performed with the use of the “COMBI 4000” (Homoth Medizinelektronik GmbH &Co KG, Baumacker 1a, 22523 Hamburg, Germany) ENT diagnostic module from Homoth Medizineelektronik, which combines the functions of a rhinomanometer and a tympanometer. The use of the HOMOTH Tymp 4000 involves applying a probe to the patient’s ear. The tip of the probe should be positioned in the external auditory canal, and the probe should fit snugly in the canal. The measurement of tympanic membrane compliance can be monitored in real-time on the display. The compliance curve (tympanometric curve) is drawn during the measurement, allowing the examiner to immediately determine impedance. The device is capable of measuring pressure values in the middle ear within the range of +200 to −400 daPa. The ear canal volume is measured in milliliters (mL). The normal volume of the ear canal should fall within the range of 0.2 mL to 3 mL.

The rhinomanometry test was conducted by placing nasal probes into the nostrils. The patient breathes naturally, and the intensity of breathing can be observed on the screen. The device displays airflow results in mL/s for inhalation and exhalation at 75, 100, and 300 Pa, separately for the left and right sides. Additionally, it provides the total airflow and relevant resistance coefficients.

Moreover, the patients were assessed with the use of the Sinonasal Outcome Test-22 Questionnaire (SNOT-22) which is widely used to examine the impact of sinonasal disease on a person’s quality of life. It consists of 22 questions that cover a range of symptoms related to the nose and sinuses. The questions are focused on various aspects, including nasal and facial symptoms, sleep disturbances, and psychological well-being. The primary purpose of the SNOT-22 is to provide a comprehensive evaluation of how sinonasal conditions affect an individual’s daily life and well-being. The questions in the SNOT-22 are categorized into different domains, such as rhinologic symptoms (e.g., nasal blockage, runny nose, need to blow nose), and ear problems such as: ear fullness, facial symptoms, and sleep disturbances or psychological issues. Each question is typically scored on a scale from 0 to 5, with 0 indicating no problem and 5 indicating a severe problem. The scores are then added up to calculate the total score, providing a quantitative measure of the impact of sinonasal disease.

The statistical analysis was performed in R statistical software, version 4.1.2. All variables were numeric or ordinal. Characteristics were presented with mean, standard deviation, and range for age due to distribution normality, and median and range for Hbd due to non-normal distribution. Distribution normality was verified with Shapiro–Wilk outcomes, skewness and kurtosis. Relationships between selected pairs of variables were analyzed with the Spearman correlation. A Repeated measures Anova was used to compare the dependance of flow parameters on pressure used for measurement. Pairwise comparisons were performed with a paired *t*-test with Bonferroni correction. All tests assumed alpha = 0.05.

The study obtained approval from the Bioethics Committee of the Regional Medical Chamber in Warsaw—registration number KB/1440/23.

## 3. Results

### 3.1. The Results of Sinonasal Conditions Evaluation

#### 3.1.1. The Subjective Assessment of Nasal Patency in SNOT-22

In the study group of 50 females, 48 patients (96%) reported a sensation of impaired nasal patency during pregnancy in the SNOT-22 questionnaire (rho = 0.77, *p* < 0.001). Additionally, the patients reported upper respiratory tract symptoms, such as the need to blow their nose (*n* = 35, 70%), sneezing (*n* = 33, 66%) and a runny nose (*n* = 31, 62%). Nasal obstruction was the most common problem, assessed on average at 2.48 ± 1.16 (minimum 0, maximum 5) and defined as strong or worse by 42% of the study group. Other nasal symptoms were noticed by 62–70% of pregnant women, but their intensity was mostly mild or moderate and assessed as the following: need to blow nose on 1.36 ± 1.27, sneezing on 1.34 ± 1.27 and runny nose on 1.06 ± 1.00. The outcomes of the SNOT-22 in the research group are presented in Table 1.

**Figure 3:**
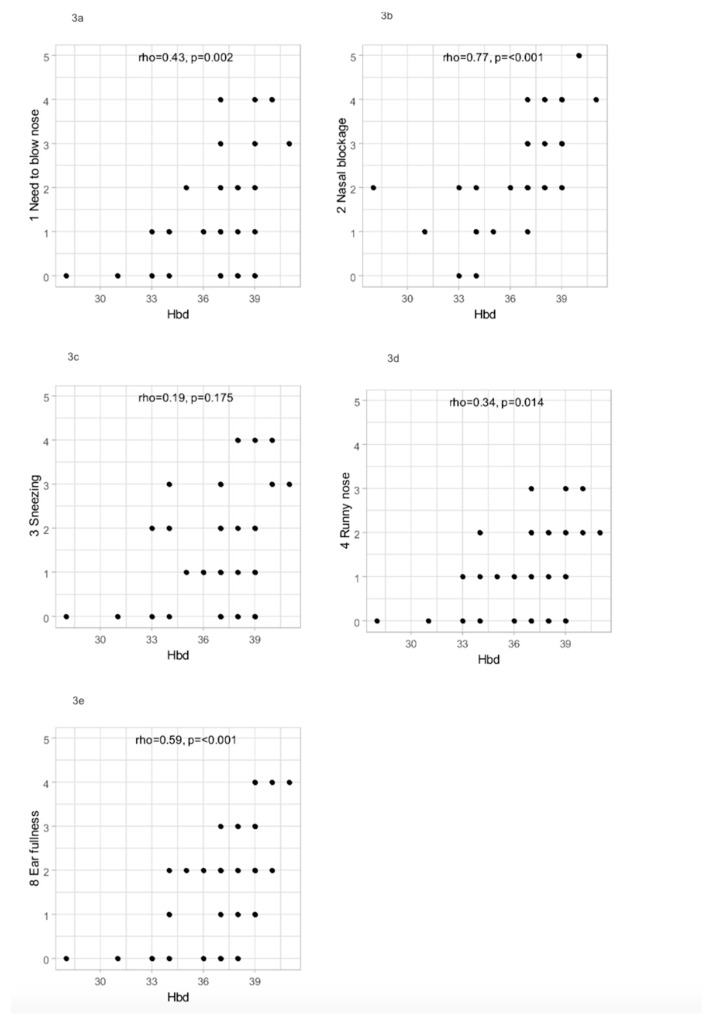
(**a**–**e**) Scatterplot presenting relationships between Hbd and selected SNOT-22 outcomes.

The severity of subjective nasal obstruction and other upper respiratory symptoms were significantly statistically correlated with the week of pregnancy. The relationship between the week of pregnancy and selected parameters was analyzed with the Spearman correlation due to the nonparametric nature of the majority of the variables. The positive relationship identified between week of pregnancy and outcomes of SNOT-22 is presented in Figure 3a–e.

In the control group of 25 females, 10 patients (40%) reported the sensation of nasal obstruction in the SNOT-22 questionnaire as mild (n = 9) and moderate (n = 1).

**Figure 4:**
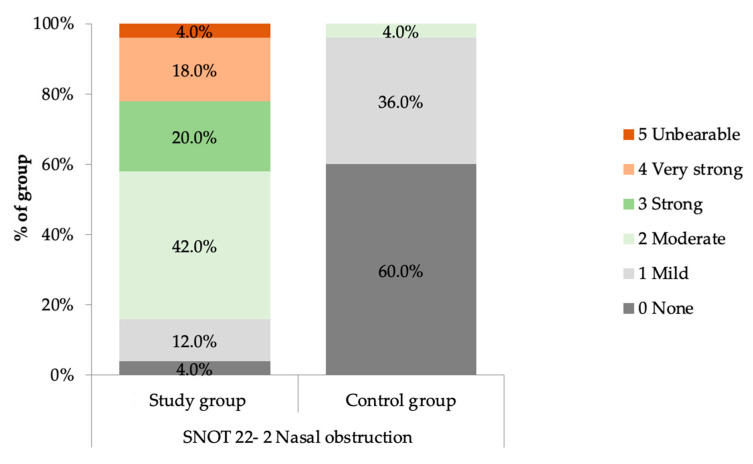
Severity of nasal obstruction in the study group and control group, based on SNOT-22 questionnaire.

The findings confirm that the severity of nasal obstruction was significantly higher in the research group than in the control group, MD = 2.04 CI_95_ (1.64; 2.44), *p* < 0.001. The severity of nasal obstruction in the research group and the control group, based on the SNOT-22 questionnaire, is presented in Figure 4.

#### 3.1.2. The Objective Assessment of Nasal Patency in Rhinomanometry

We found that in the rhinomanometry of the study group the minimum airflow was 0 mL/s and the maximum airflow was 487 mL/s (exsp 75Pa L). In the study group, the dependence of the level of the rhinomanometry parameters on the pressure used with the measurement turned out to be significant, with a value of *p* < 0.001 for all parameters. For each parameter, the flow decreased significantly, with increasing pressure (Table 2).

We observed negative relationships between the week of pregnancy and the rhinomanometry outcome. The strength of the correlations for all analyzed parameters were moderate or high (rho ranged from −0.82 to −0.60, depending on parameter, *p* < 0.001 for all parameters). Higher Hbd was associated with lower flow.

In the control group the minimum airflow was 98.00 mL/s (insp 300 Pa L) and the maximum airflow was 436 mL/s (insp 300 Pa L). The findings confirm that, in control group, dependence of rhinomanometry parameters’ level on the pressure used with measurement turned out to not be significant for any parameter (*p* > 0.05), Table 2.

Moreover, comparing results in both groups, we observed that severity of nasal obstruction was significantly higher in research group than in control group (MD = 2.04 CI_95_ (1.64;2.44), *p* < 0.001).

Relationship between subjective (SNOT-22 question 2: nasal obstruction) and objective (rhinomanometry outcome) assessment of nasal patency in the study group was analysed with Spearman correlation due to nonparametric nature of variables. The data suggest that, for all rhinomanometry parameters negative relationships with subjective nasal obstruction were identified (rho ranged from −0.69 to −0.62, depending on the parameter, with *p* < 0.001 for all parameters). The higher subjective nasal obstruction was associated with a lower flow based on rhinomanometry and the strength of the relationships was moderate/high.

### 3.2. The Results of the Eustachian Tube Evaluation

#### 3.2.1. The Subjective Assessment of the Eustachian Tube Patency in SNOT-22

In the SNOT-22 survey, 34 patients (68%) out of the 50 in the study group reported a subjective sensation of congested ears (36%—moderate, 12%—strong, 8%—very strong). A higher week of pregnancy was associated with higher ear fullness (rho = 0.59, *p* < 0.001) (Figure 3e). Simultaneously, in the control group, nine patients (36%) out of 25 reported a subjective sensation of congested ears. Each of them rated the ear problem as one point, so “mild”. Statistical analysis showed that the severity of ear blockage was significantly higher in the research group than in the control group (MD = 2.00 CI_95_ (1.00;2.00), *p* < 0.001).

#### 3.2.2. The Objective Assessment of the Eustachian Tube Patency in Tympanometry

In the study group of 50 women, the lowest value in tympanometry was −138 daPa, while the highest was 22 daPa. All women exhibited a type A tympanogram in the tympanometric examination. The average value of tympanometry in the left ear was −60.22 ± 33.79 (daPa), and −61.68 ± 32.07 (daPa) in the right ear. Despite the presence of only a type A tympanogram, tympanometry values decreased with higher weeks of pregnancy.

Moreover, a negative relationship was identified between Hbd with tympanometry results for both ears (PEAK L/R with high strength, rho = −0.85, *p* < 0.001,) which meant that higher Hbd was associated with lower PEAK L/R and the strength of the relationship was high (Table 2). The data suggested a correlation between the advancement of pregnancy and the objective (tympanometric) assessment of Eustachian tube patency.

In the control group, the lowest value in tympanometry was −26 daPa while the highest was 51 daPa. A total of 25 women exhibited a type A tympanogram in the tympanometric examination. The average value of tympanometry in the left ear was 9.48 ± 22.45 (daPa), and 8.72 ± 23.99 (daPa) in the right ear.

**Figure 5:**
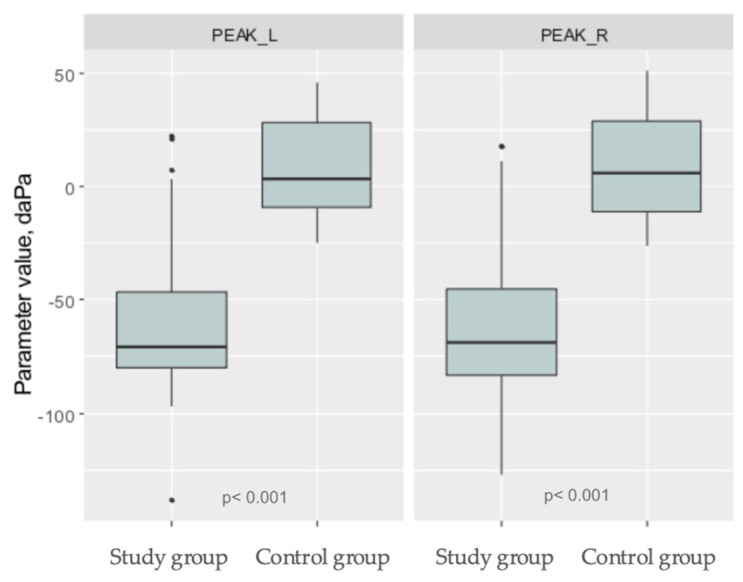
Boxplots representing level of analyzed tympanometry parameters in research group and control group. PEAK L- parameter value in left ear. PEAK R- parameter value in right ear.

Statistical analysis confirmed a significant difference between the study group and the control group in the tympanometry for both ears (*p* < 0.001) (Figure 5).

A significant correlation between the subjective and objective assessment of the Eustachian tube patency was observed in both groups. Patients with no ear blockage had positive PEAK L/R level, while patients with an ear blockage had negative PEAK L/R level (*p* < 0.001).

## 4. Discussion

The physiological fluctuations in estrogen and progesterone levels play a crucial role in shaping the manifestations observed in pregnant women, ranging from nasal obstruction to Eustachian tube dysfunction [14,15]. The presented study confirmed that hormonal changes during pregnancy impact the upper respiratory tract, particularly the nasal cavity and Eustachian tube, and this observation remains in line with other authors [1,16,17,18,19].

Our findings demonstrate a high incidence of subjective nasal obstruction in the study group (96%), assessed by 42% as strong or worse. Most authors reported subjective nasal congestion in a lower percentage of patients, probably because they assessed women throughout the entire pregnancy in each trimester, while we examined patients only in the third trimester, when these symptoms are most pronounced [20,21,22]. Additionally, the objective assessment of nasal patency through rhinomanometry revealed a significant decrease in airflow with increasing pressure in the study group and indicated a progressive decline in nasal patency as pregnancy advanced. Demir et al. also reported that pregnancy has a negative impact on nasal physiology, causing breathing difficulties in certain women and measurable changes in nasal parameters [23].

Moreover, similar to other authors, we noticed that other nasal symptoms, such as the need to blow the nose, sneezing, and a runny nose, were also correlated significantly with the week of pregnancy [18,24,25,26]. To compare, Baudoin et al. [21] noticed subjective symptoms of PIR (Pregnancy-induced rhinitis) in 31.86%. The median duration of PIR was four months, with a complete resolution of symptoms between 2th and 16th day after delivery in the majority of respondents. In the study, the clinical presentation of pregnancy rhinitis included nasal obstruction as the most common symptom, followed by rhinorrhea, postnasal secretion, nose itching, sneezing, and hyposmia.

Our objective measurement aligns with the subjective reports of nasal obstruction, providing a comprehensive understanding of the physiological changes affecting the upper respiratory tract during pregnancy. These observations raise questions about the safe and effective management of otorhinolaryngological manifestations in pregnant women. The awareness of the impact on the quality of life for pregnant women is also crucial for healthcare practitioners and gynecologists, enabling them to anticipate, assess, and manage symptoms adequately.

Medical treatment options for patients with rhinitis during pregnancy need to be carefully considered. Especially in cases of severe nasal symptoms, therapy involving nasal saline mist, antihistamines, and topical corticosteroids is recommended [27,28]. Some investigators report that intranasal corticosteroid injections are also useful, but must be administered under expert care [29].

In our research, the results of pregnant women were compared to the control group consisting of 25 healthy non-pregnant women and medical students. Similar comparisons was conducted by Philpot et al. [30]. The study established a significant difference in the severity of nasal obstruction between the pregnant and non-pregnant groups, emphasizing the unique challenges posed by hormonal fluctuations during pregnancy [30]. The available research findings indicate notable alterations in both the structure and function of the nasal mucosa in pregnancy [31]. In contrast, Mabry’s research implies that nasal congestion could not be explained only by estrogen, because the emotional factors might significantly contribute to how symptoms from the nose are perceived.

The second aspect discussed in the study was the impact of pregnancy on the Eustachian tube patency. We explored the Eustachian tube function, both subjectively through the SNOT-22 questionnaire and objectively through tympanometry. A total of 68% of pregnant women reported a sensation of congested ears. However, the objective assessment of the Eustachian tube patency in tympanometry showed that all pregnant women had a type A tympanogram. The same observation was made Tsunoda et al., who conducted pure-tone audiometry and tympanometry in a group of 56 pregnant women and found the hearing to be normal in all [32]. However, it should be emphasized that in our study the tympanometry values decreased with higher weeks of pregnancy, and statistical analysis confirmed a significant difference between the study group and control group in tympanometry for both ears. This observation may explain the high rate of subjective ear discomfort reported by pregnant women.

This observation is consistent with other authors. Kwatra et al. [33] also reported that in their study, a significant difference was seen between the average of air conduction threshold values in speech frequencies when antepartum values were compared with postpartum values.

The results obtained by some authors [15,33], showed that there is a decrease in low frequency hearing level during pregnancy, similar to Meniere disease, but in pregnancy, the values remain within normal limits. The cause of the abovementioned change can be due to salt and water retention, which is seen during pregnancy. There have been studies in past changes in hearing threshold values in pregnancy, such as sudden sensorineural hearing loss in an uncomplicated pregnancy. It was probably caused by the hypercoagulable state during pregnancy, leading to vascular occlusion in the inner ear [34,35,36].

To sum up, the main observation of our study was a statistically important decrease in the tympanometry values of the study group compared to the control group, which may explain the hearing problems in the pregnant women despite the presence of tympanometry A. This observation is consistent with the worse rhinomanometry results obtained from pregnant women. The subjective experiences of the women correspond with the objective research findings.

It is imperative to acknowledge the limitations of the study, including the exclusion of severe pathologies that could influence nasal patency. Future research endeavors may explore the nuances of hormonal variations in different trimesters and their specific contributions to otorhinolaryngological manifestations. Future studies could further enhance our understanding of the persistence or resolution of postpartum symptoms.

## 5. Conclusions

In conclusion, this study significantly contributes to our understanding of the complex interplay between hormonal changes and physiological alterations in the upper respiratory tract during pregnancy. The results highlight the substantial impact on both subjective and objective measurements of nasal and Eustachian tube patency. Almost every woman in the third trimester of pregnancy experiences nasal obstruction, and nearly 70% struggle with ear fullness. The nose- and ear-related symptoms commonly experienced during pregnancy often result from changes in sex hormones. Fortunately, most of these symptoms can be managed with non-invasive treatments, as they tend to resolve on their own after childbirth. Recognizing and addressing these challenges is pivotal for ensuring the well-being of pregnant individuals and enhancing the quality of antenatal care. These increasingly recognized conditions have become significant, not only because of their link to maternal obstructive sleep apnea (OSAS) and potential adverse outcomes for the fetus, but also due to their notable impact on the quality of life for pregnant women. It is essential to consider the safety profile and current evidence supporting the available interventions and medications. More research with larger groups is needed to confirm and better understand the accuracy of these discoveries.

## Figures and Tables

**Table 1:** Outcomes of SNOT-22 in research group (*n* = 50).

Variable	Number (%) of Patients with a Given Severity of Symptoms (0—None, 1—Mild, 2—Moderate, 3—Strong, 4—Very Strong, 5—Unbearable)					
	0	1	2	3	4	5
1 Need to blow nose	15 (30%)	16 (32%)	10 (20%)	4 (8%)	5 (10%)	0 (0%)
2 Nasal obstruction	2 (4%)	6 (12%)	21 (42%)	10 (20%)	9 (18%)	2 (4%)
3 Sneezing	17 (34%)	12 (24%)	12 (24.0)	5 (10%)	4 (8%)	0 (0%)
4 Runny nose	19 (38%)	13 (26%)	14 (28.0)	4 (8%)	0 (0%)	0 (0%)

**Table 2:** Association between measurement pressure and level of rhinomanometry outcome in the study group and control group.

	STUDY GROUP				CONTROL GROUP			
Variable	75 Pa	150 Pa	300 Pa	*p*	75 Pa	150 Pa	300 Pa	*p*
Rhinomanometry								
insp L, mL/s	271.02 ± 76.26	251.62 ± 75.31	205.04 ± 92.71	<0.001	258.56 ± 70.59	271.04 ± 71.15	266.20 ± 85.65	0.387
exsp L, mL/s	261.60 ± 91.68	234.90 ± 92.99	182.58 ± 98.64	<0.001	238.16 ± 48.23	241.72 ± 47.81	241.84 ± 66.92	0.860
insp R, mL/s	234.02 ± 92.66	217.62 ± 86.17	180.30 ± 98.97	<0.001	260.44 ± 49.36	262.32 ± 54.73	271.84 ± 67.29	0.313
exsp R, mL/s	234.80 ± 88.28	216.24 ± 82.25	164.40 ± 97.66	<0.001	255.96 ± 52.33	246.08 ± 63.51	238.48 ± 68.21	0.097

## Data Availability

All subjects gave their informed consent for inclusion before they participated in the study. The study was conducted in accordance with the Declaration of Helsinki, and the protocol was approved by the Bioethics Committee of the Regional Medical Chamber in Warsaw—registration number KB/1440/23.
